# Does self-reported pregnancy loss identify women at risk of an adverse cardiovascular phenotype in later life? Insights from UK Biobank

**DOI:** 10.1371/journal.pone.0223125

**Published:** 2019-10-23

**Authors:** Einas Elmahi, Mihir M. Sanghvi, Alexander Jones, Christina Y. L. Aye, Adam J. Lewandowski, Nay Aung, Jackie A. Cooper, José Miguel Paiva, Elena Lukaschuk, Stefan K. Piechnik, Stefan Neubauer, Steffen E. Petersen, Paul Leeson

**Affiliations:** 1 Oxford Cardiovascular Clinical Research Facility, Radcliffe Department of Medicine, Division of Cardiovascular Medicine, Radcliffe Department of Medicine, University of Oxford, John Radcliffe, Oxford, United Kingdom; 2 William Harvey Research Institute, NIHR Biomedical Research Centre at Barts, Queen Mary University of London, Charterhouse Square, London, Unite Kingdom, Barts Heart Centre, St Bartholomew’s Hospital, Barts Health NHS Trust, West Smithfield, London, United Kingdom; 3 Department of Paediatrics, Children’s Hospital, John Radcliffe, University of Oxford, Oxford, United Kingdom; 4 The Nuffield Department of Women's & Reproductive Health, Medical Science Division, University of Oxford, Oxford, United Kingdom; 5 Oxford Centre for Clinical Magnetic Resonance Research, Division of Cardiovascular Medicine, Radcliffe Department of Medicine, University of Oxford, Oxford, United Kingdom; Medical University Innsbruck, AUSTRIA

## Abstract

**Introduction:**

Cardiovascular disease (CVD) is more common in women who have had pregnancy complications such as spontaneous pregnancy loss. We used cross-sectional data from the UK Biobank Imaging Enhancement Study to determine whether pregnancy loss is associated with cardiac or vascular remodelling in later life, which might contribute to this increased risk.

**Methods:**

Pregnancy history was reported by women participating in UK Biobank between 2006 and 2010 at age 40–69 years using a self-completed touch-screen questionnaire. Associations between self-reported spontaneous pregnancy loss and cardiovascular measures, collected in women who participated in the Imaging Enhancement Study up to the end of 2015, were examined. Cardiac structure and function were assessed by magnetic resonance (CMR) steady-state free precession imaging at 1.5 Tesla. Carotid intima-media thickness (CIMT) measurements were taken for both common carotid arteries using a CardioHealth Station. Statistical associations with CMR and carotid measures were adjusted for age, BMI and other cardiovascular risk factors.

**Results:**

Data were available on 2660 women of whom 111 were excluded because of pre-existing cardiovascular disease and 30 had no pregnancy information available. Of the remaining 2519, 446 were nulligravid and 2073 had a history of pregnancies, of whom 622 reported at least one pregnancy loss (92% miscarriages and 8% stillbirths) and 1451 reported no pregnancy loss. No significant differences in any cardiac or carotid parameters were evident in women who reported pregnancy loss compared to other groups (Table 1).

**Conclusion:**

Women who self-report pregnancy loss do not have significant differences in cardiac structure, cardiac function, or carotid structure in later life to explain their increased cardiovascular risk. This suggests any cardiovascular risks associated with pregnancy loss operate through other disease mechanisms. Alternatively, other characteristics of pregnancy loss, which we were not able to take account of, such as timing and number of pregnancy losses may be required to identify those at greatest cardiovascular risk.

## Introduction

Spontaneous pregnancy loss is the most common and least studied complication of pregnancy, with 32% of all conceptions (clinically and non-clinically apparent pregnancy loss) resulting in loss of the fetus and about 15% of all clinically-recognized pregnancies failing to survive to delivery [1, 2]. A history of pregnancy loss (miscarriage and stillbirth) is linked to coronary artery disease (CAD) in later life[3]. In a meta-analysis of ten cohort and case-control studies, women with a history of a single pregnancy loss were reported to have 45% increased risk of CAD but not of other CVD or stroke, while the risk in women with recurrent pregnancy loss was doubled [4, 5]. Women who had more than three miscarriages had a nine-fold greater risk of myocardial infarction (MI) and those who had a stillbirth were nearly three times as likely to experience a coronary disease event [6]. The risk of future MI associated with pregnancy loss appears to be independent of other atherosclerotic cardiovascular disease (ASCVD) risk factors and directly proportional to the number of pregnancy losses [6, 7]. Deeper understanding of mechanisms that underpin these associations is therefore required if optimal ways to protect the future cardiovascular health of those who suffer pregnancy loss are to be identified[8].

Non-invasive measures of cardiovascular structure and function have demonstrable value in prediction of future CVD risk and are not entirely determined by the existence of traditional risk factors, such as hypertension. Abnormal cardiovascular magnetic resonance (CMR) measures of left ventricular (LV) geometry and LV hypertrophy (LVH) describe a cardiac phenotype that adds supplemental risk to that predicted by traditional cardiovascular risk factors [9]. Carotid intima-media thickness (CIMT) provides an assessment of the severity of vascular wall changes associated with an individual risk profile. Therefore, we used these early markers of cardiovascular risk to investigate whether pregnancy loss was associated with a high-risk cardiovascular phenotype in a large cross-sectional cohort of women from the UK Biobank Imaging Enhancement Study. The hypothesis was that the known association of pregnancy loss with cardiovascular disease risk, which is not explained by traditional risk factors, might be in part explained by abnormal cardiac and vascular re-modelling.

## Material and methods

### Ethical approval

The UK Biobank study has been approved by the—North West—Haydock Research Ethics Committee (REC reference: 16/NW/0274). The committee gave a favourable ethical opinion of the UK Biobank research and has also confirmed that the favourable ethical opinion applies to all research projects conducted in the UK using tissue or data supplied by the tissue bank, provided that the release of the tissue or data complies with the committee’s specific conditions.

### Study population

**Fig 1:**
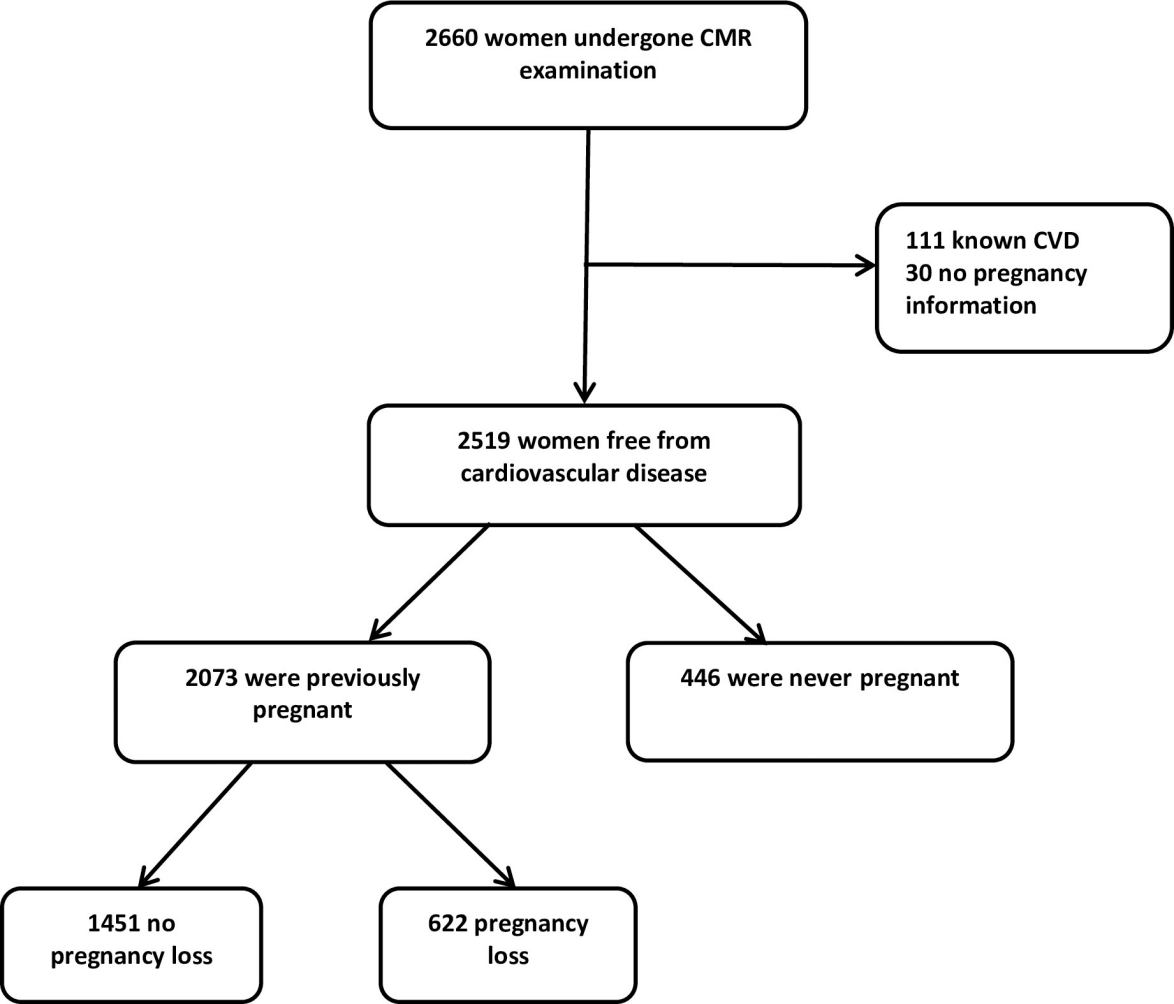
Case selection flowchart.

We studied 2,660 women, aged 40–69 years, from the UK Biobank Imaging Enhancement Study population (Fig 1). These women were assessed in 22 UK centres to provide socioeconomic and ethnic heterogeneity and an urban-rural mix. The UK Biobank study protocol is described in detail elsewhere [10].

As the aim of this study was to see whether pregnancy loss is associated with subclinical changes in cardiovascular structure and function i.e. before the development of CVD, women with a prior diagnosis of CAD were excluded from the analysis. All participants were asked to sign a written informed consent for imaging assessment.

Information on material deprivation, social deprivation, socioeconomic class and education were collected using the touch-screen self-administered questionnaire. Other information such as smoking and alcohol consumption, medical and reproductive history (including pregnancy history and any history of spontaneous pregnancy loss or stillbirth) were also included in the questionnaire, along with questions that allowed participants to be ranked according to their level of physical activity (vigorous, moderate and walking).

Baseline physical measurements were taken. Blood pressure (BP) was measured twice (a minute apart) using the Omron HEM-7015IT digital blood pressure monitor (recommended by the British Hypertension Society). Weight and height were measured using Tanita BC-418 MA body composition analysers and Seca 202 height measures, respectively.

The women were categorised into three groups, according to their reproductive history: (1) women with a history of pregnancy without loss; (2) women with a history of pregnancy loss; and (3) nulligravid women.

### CMR imaging protocol and analysis

Each participant underwent a CMR protocol without pharmacological stressor or contrast agent. Cardiac measurements were made using balanced steady-state free precession (bSSFP) CMR imaging at 1.5 Tesla (MAGNETOM Aera, Syngo Platform VD13A, Siemens Healthcare, Erlangen, Germany) [11] that included a complete short axis (SA) stack of bSSFP cines to allow assessment of volumes left ventricular function. The analysis in this study utilizes the manual analysis by two independent core labs according to standardized processing guidelines (https://jcmr-online.biomedcentral.com/articles/10.1186/s12968-017-0327-9).

### CIMT scanning protocol

Ultrasound images of the common carotid arteries were obtained in short and long axes from low in the neck up to the jaw, at least to the level of the carotid bifurcation using a CardioHealth Station (Panasonic Healthcare Corporation of North America, Newark, NJ, 19 USA), with a 9MHz linear array transducer. CIMT was measured at two angles for each carotid (150^o^ /120^o^ right and 210^o^/240^o^ left) and the mean, maximum and minimum CIMT tracking for each carotid was recorded [12].

### Statistical analysis

Prior to analysis, all dependent variables were assessed for normality using histograms and quartile-quartile plots. Natural logarithmic transformation was used where necessary to satisfy assumptions of normality. Outliers were defined as measurements more than three interquartile ranges below the first quartile or above the third quartile and were excluded.

We examined the impact of pregnancy loss, being pregnant without any previous miscarriages and never being pregnant on cardiac structure and function using regression models fitted for each cardiac (dependent) variable. Results are presented unadjusted and then adjusted by multiple regression for age, ethnicity, height, body mass index, systolic blood pressure, diastolic blood pressure, smoking, alcohol use, self-reported raised cholesterol, presence of diabetes, Townsend deprivation score, income, qualifications, live births, aspirin use, and physical activity as determined by accelerometer data. We used the same independent covariates in models examining relationships between pregnancy status and left-sided CIMT, right-sided CIMT and average CIMT.

Differences in means between groups were assessed by t-test or ANOVA, as appropriate, and differences in percentages were assessed using chi-squared tests.

The total proportion of data missing is 3.7% and 47.5% of the participants have missing data on at least one variable. Multiple imputations by chained equations (MICE) were used to impute missing data in 60 datasets, as follows. Predictive mean matching with five nearest neighbours was used for continuous variables and logistic regression for binary variables. All variables used in the analysis models were included in the imputation. Plots were examined to assess convergence and plausibility of estimates. The analysis was run on the 60 individual datasets and the results were pooled. The fraction of missing information for the pregnancy coefficients ranges from 0.009 (LVEF) to 0.088 (LAMaxV) indicating low variability between imputed datasets i.e. the observed data is providing adequate information about the missing data (Madley-Dowd et al.).

Descriptive statistics for continuous variables were presented as mean ± standard deviation or median and interquartile range (IQR), with categorical variables presented as number (percentage).

The ANOVA p values for CMR and CIMT measures were calculated from the F-statistics and reflect the overall between-group variability.

## Results

### Study population

Demographics for women with a history of pregnancy without loss (n = 1451), those with a history of pregnancy loss (n = 662), and nulligravidae (n = 446) are presented in Table 1 compared to nulligravidae. Women with a history of pregnancy were older, less educated and had a lower socioeconomic class. The significant difference in systolic blood pressure detected between all groups did not remain after adjustment for age and body mass index (BMI).

**Table 1:** Participant characteristics.

Variable	No History of Pregnancy Loss	History of Pregnancy Loss	Nulligravid	*P*
	(n = 1451 [57%])	(n = 622 [24.7%])	(n = 446 [17.7%])	^(ANOVA)^
**BMI** (Kg/m^2^)	26.1 ± 4.5	26.5 ± 4.6	26.1 ± 5.0	0.1
**Systolic BP** (mmHg)	134.0 ± 18.8	134.0 ± 19.1	129.4 ± 17.4	<0.001
**Diastolic BP** (mmHg)	77.6 ± 9.8	77.5 ± 10.0	76.6 ± 9.8	0.2
**Hypercholesterolemia** (Y/N)	14.1% (204)	15.4% (96)	12.8% (57)	0.5
**Diabetes** (Y/N)	3.2% (46)	3.5% (22)	4.9% (22)	0.2
**Caucasian Ethnicity** (Y/N)	97.8% (1419)	96.6% (601)	97.5% (435)	0.3
**Height** (cm)	163.1 (6.5)	163.0 (6.1)	164.6 (6.9)	<0.001
**Current smoker** (Y/N)	3.3% (47)	4.1% (25)	3.6% (16)	0.7
**Regular alcohol** (Y/N)	37.7% (541)	37.1% (229)	40.3% (179)	0.5
**Accelerometery score** (mean vector magnitude)	28.4 ± 8.5	28.7 ± 9.6	28.4 ± 9.0	0.8
**Townsend score**	-2.10 ± 2.63	-2.0 ± 2.6	-1.33 ± 2.8	<0.001
**Annual household income** (£)				
**<18,000**	16.8% (226)	16.5% (96)	?	
**18–30,999**	30.4% (409)	29.4% (171)	?	?
**31–51,999**	28.0% (377)	29.7% (173)	?	
**52–100,000**	19.2% (258)	18.0% (105)	?	
**>100,000**	5.6% (76	6.4% (37)	?	
**Qualifications** (degree/professional)	58.7% (851)	-2.0 ± 2.6	??????	?
**Aspirin use** (Y/N)	4.8% (70)	-2.0 ± 2.6	17.8% (76)	0.6
**Number of live births—**Median (IQR)	2 (2–3)	-2.0 ± 2.6	31.9% (136)	<0.001
**Number of live births—**Median (IQR)	0 (0–0)	-2.0 ± 2.6	29.7% (127)	0.95

### Cardiac parameters measured using CMR imaging

Cardiac indices measured for all groups are presented in Table 2. No significant differences were detected in CMR parameters between the groups after adjustment for potential confounders (Table 3).

**Table 2:** Unadjusted CMR cardiac geometry.

Variable	Pregnancy Status	Means ± SE	P value
			(ANOVA)
			* *
**LVEDV** (ml)	**Pregnancy Loss**	**124.0 ± 22.2**	** **
	**No pregnancy**	**125.9 ± 21.9**	**0.075**
	**Pregnancy (no loss)**	**123.1 ± 22.7**	
**LVESV** (ml)	**Pregnancy Loss**	**48.5 ± 12.1**	** **
	**No pregnancy**	**49.5 ± 12.0**	**0.017**
	**Pregnancy (no loss)**	**47.6 ± 12.5**	
	**LVSV** (ml)	**Pregnancy Loss**	**74.5 ± 14.4**	** **
**No pregnancy**		**75.6 ± 14.1**	**0.363**
**Pregnancy (no loss)**		**74.5 ± 14.4**	
**LVEF** (%)		**Pregnancy Loss**	**60.4 ± 6.0**	** **
	**No pregnancy**	**60.4 ± 5.6**	**0.177**
	**Pregnancy (no loss)**	**60.9 ± 5.9**	
	**LVM** (g)	**Pregnancy Loss**	**73.0 ± 14.1**	** **
**No pregnancy**		**72.4 ± 13.7**	**0.501**
**Pregnancy (no loss)**		**72.2 ± 14.2)**	
**LAMaxV** (ml)		**Loss**	**60.8 ± 16.5**	** **
	**No pregnancy**	**62.5 ± 18.1**	**0.051**
	**Pregnancy (no loss)**	**60.1 ± 18.3**	
	**LV Mass: Volume**	**Loss**	**0.59 ± 0.10**	** **
** ** (Ratio in g/ml)	**No pregnancy**	**0.58 ± 0.10**	**0.079**
	**Pregnancy (no loss)**	**0.59 ± 0.10**	

**Table 3:** CMR cardiac geometry adjusted for potential confounders.

Variable	Pregnancy Status	Means ± SE	Effect Size (%)	95% CI	*P*
**LVEDV** (ml)	**Pregnancy Loss**	**122.1 ±1.0**	**0.9**	**(-1.52, 1.37)**	**0.91**
	**No pregnancy**	**124.1 ± 1.4**	**1.59**	**(-0.64, 3.86)**	**0.16**
	**Pregnancy (no loss)**	**122.2 ± 0.8**	**0**	**-**	**-**
	**LVESV** (ml)	**Pregnancy Loss**	**47.8 ± 0.6**	**1.05**	**(-1.19, 3.27)**	**0.36**
**No pregnancy**		**48.0 ± 0.8**	**1.55**	**(-1.84, 5.06)**	**0.37**
**Pregnancy (no loss)**		**47.3 ± 0.5**	**0**	**-**	**-**
**LVSV** (ml)		**Pregnancy Loss**	**73.5 ± 0.7**	**-0.68**	**(-2.24, 0.91)**	**0.4**
	**No pregnancy**	**75.2 ± 0.9**	**1.72**	**(-0.70, 4.20)**	**0.17**
	**Pregnancy (no loss)**	**74.0 ± 0.5**	**0**	**-**	**-**
	**LVEF** (%)	**Pregnancy Loss**	**60.5 ± 0.3**	**-0.42**	**(-0.99, 0.15)**	**0.15**
**No pregnancy**		**60.9 ± 0.4**	**0.02**	**(-0.85, 0.88)**	**0.97**
**Pregnancy (no loss)**		**60.9 ± 0.3**	**0**	**-**	**-**
**LVM** (g)		**Pregnancy Loss**	**70.6 ± 0.6**	**0.22**	**(-1.32, 1.79)**	**0.78**
	**No pregnancy**	**70.5 ± 0.8**	**0.16**	**(-2.18, 2.55)**	**0.9**
	**Pregnancy (no loss)**	**70.4 ± 0.5**	**0**	**-**	**-**
	**LAMaxV** (ml)	**Pregnancy Loss**	**58.7 ± 0.9**	**-0.17**	**(-2.75, 0.90)**	**0.9**
**No pregnancy**		**61.1 ± 1.2**	**3.91**	**(-0.05, 8.02)**	**0.053**
**Pregnancy (no loss)**		**58.8 ± 0.7**	**0**	**-**	**-**
**LV Mass: Volume** (Ratio in ml/g)		**Pregnancy Loss**	**0.58 ± 0.005**	**0.31**	**(-1.31, 1.96)**	**0.71**
	**No pregnancy**	**0.57 ± 0.007**	**-1.41**	**(-3.81, 1.06)**	**0.26**
	**Pregnancy (no loss)**	**0.58 ± 0.004**	**0**	**-**	**-**

CMR parameters adjusted for age, ethnicity, height, BMI, SBP, DBP, smoking, alcohol, raised cholesterol, diabetes, Townsend score, income, qualifications, live births, aspirin use, exercise (accelerometer).

### CIMT measurements

Table 4 shows CIMT unadjusted measurements for both carotids (taken at different angles) for all groups with no significant differences identified between the groups before or after adjustment for potential confounders (Table 5).

**Table 4:** Unadjusted CIMT measurements.

Carotid Artery (measurement angle)	Pregnancy Status	Unadjusted means (SE)	Effect size (%)	95% confidence	*P*
**Left** (120^o^, 150^o^)	**Pregnancy Loss**	**635.4 ± 7.5**	**1.1**	**-0.97, 3.22**	**0.301**
	**No pregnancy**	**615.6 ± 9.7**	**-2.05**	**-5.05, 1.04**	**0.191**
	**Pregnancy (no loss)**	**628.4 ± 5.6**	**0**	**-**	**-**
**Right** (210^o^, 240^o^)	**Pregnancy Loss**	**631.3 ± 7.7**	**0.9**	**-1.21, 3.06**	**0.405**
	**No pregnancy**	**623.0 ± 9.9**	**-0.37**	**-3.49, 2.84**	**0.818**
	**Pregnancy (no loss)**	**625.8 ± 5.7**	**0**	**-**	**-**
	**Average measures**	**Pregnancy Loss**	**633.3 ± 6.5**	**1**	**-0.8, 2.83**	**0.277**
**No pregnancy**		**619.3 ± 8.4**	**-1.22**	**-3.85. 1.49**	**0.375**
**Pregnancy (no loss)**		**627.1 ± 4.9**	**0**	**-**	**-**

**Table 5:** CIMT measurements adjusted for potential confounders.

Carotid Artery	Pregnancy Status	Means (SE)	*P*
(measurement angle)			(ANOVA)
**Left** (120^o^, 150^o^)	**Pregnancy Loss**	**640.9 (102.1)**	** **
	**No pregnancy**	**619.0 (96.2)**	**0.066**
	**Pregnancy (no loss)**	**636.2 (102.0)**	
	**Right** (210^o^, 240^o^)	**Pregnancy Loss**	**640.7 (111.6)**	** **
**No pregnancy**		**627.1 (98.0)**	**0.415**
**Pregnancy (no loss)**		**636.2 (105.2)**	
**Average measures**		**Pregnancy Loss**	**638.1(96.3)**	** **
	**No pregnancy**	**621.7(85.6)**	**0.201**
	**Pregnancy (no loss)**	**632.5(91.0)**	

Associations adjusted for age, ethnicity, height, BMI, SBP, DBP, smoking, alcohol, raised cholesterol, diabetes, Townsend score, income, qualifications, live births, aspirin use, exercise (accelerometer).

## Discussion

To our knowledge, this is the first study to investigate the relationship between self-reported pregnancy loss and maternal cardiovascular structure and function. Women with a greater burden of pregnancy loss have been found to have increased CVD risk in later life. In this large population of CVD-free women, we found no associations between pregnancy loss and later measures of cardiovascular geometry or function to explain that risk.

It has been proposed that pregnancy loss and CVD share a common pathophysiological mechanism and genetic predisposition and this suggestion has been supported by the observation that parents of women with recurrent pregnancy loss have a higher incidence of CAD [13].

Pregnancy loss and CVD also share common risk factors such as insulin resistance[14] and chronic kidney disease[15]. Endothelial dysfunction is also central to the development of atherosclerosis[16] and Germain et al. (2006) [17] hypothesised that endothelial dysfunction caused placentation defects in women who experienced recurrent pregnancy loss. The persistence of this dysfunction after a complicated pregnancy has therefore be proposed as a potential link with future cardiovascular events and recurrent pregnancy loss has been associated with lower endothelium-dependent as well as endothelium-independent vasodilatation. These observations have led some to postulate that women with a history of pregnancy loss may have underlying cardiovascular, microvascular, and/or homeostatic dysfunctions, which in turn lead to pregnancy complications during reproductive years and CAD in later life.

Therefore, it is reasonable to expect that women with a history of pregnancy loss might exhibit subclinical cardiac remodelling that influences their cardiovascular mortality and morbidity.

Some measures of cardiac remodelling, such as increased left ventricular mass (LVM), are known risk factors for CVD and adverse cardiovascular events [18, 19]. For example, Levy D et al [20] demonstrated that LVM increments of 50g/m (even within normal limits for mass) are associated with 1.6x greater relative risk of CVD in women. Such LVM increases raise myocardial oxygen demand and reduce coronary blood supply reserve, resulting in supply-demand mismatch, which is associated with a higher risk of CAD. LVM normalised to cardiac volume (as a ratio) has been strongly associated with coronary events in asymptomatic individuals who are free of clinically apparent CVD [21]. Similarly, increased left atrial volume (LAV) in the context of sinus rhythm is a marker of left ventricular diastolic dysfunction and an established risk factor for CVD.

These markers of cardiac remodelling and function were measured using CMR, which is the gold standard technique for evaluating cardiac structure and function and detecting subclinical disease [22, 23]. Despite this, we detected no association of either pregnancy or pregnancy loss with cardiac geometry or function, when compared to a control group of similar women who had not been pregnant.

The incidence of failed pregnancies prior to becoming clinically recognised (diagnosed by urinary HCG) is far more than the incidence of spontaneous pregnancy loss in clinically recognised pregnancies[24–26]. As we used recall, women who experienced early pregnancy loss without a clinically recognised pregnancy, may have underreported very early pregnancy loss, which could have diluted associations with cardiac geometry. However, the evidence of association between pregnancy loss and higher risk of future CVD described in previous studies is also based on pregnancy loss reported by the women themselves.

Furthermore, early pregnancy loss is common [27] and its aetiology is usually different to that of mid and late trimester losses, being more commonly due to fetal chromosomal defects than due to abnormal placentation, for example [28]. Therefore, the overall impact of pregnancy loss on cardiac and vascular structure and function in this cohort could be diluted by the presence of other mechanisms unrelated to adverse cardiovascular outcomes.

We also measured carotid intima-media thickness (CIMT), a subclinical marker of vascular ageing / atherosclerosis that has been shown to reflect CVD risk profile and predict risk of future disease[29]. However, we were unable to demonstrate any significant differences between groups in CIMT measured at different angles. Diluted

### Limitations

Pregnancy loss-timing (in gestational weeks) was not available making it impossible to examine the effect of the different stages of pregnancy loss (early and late) separately in this study, this may have diluted our ability to detect effects of, for example, vascular dysfunction driving both late pregnancy loss (through abnormal placentation) and later maternal cardiac remodelling and vascular disease.

There is a higher risk of CVD in women with any history of pregnancy loss but, this risk is substantially higher in women with a history of more than three losses. The number of women with higher than 3 pregnancy losses in our cohort was small and a specific impact of a high number of pregnancy losses could not be studied.

Also, there was a lack of some reproductive information which could potentially impact the incidence of CVD in women such as, early menarche, contraception use, infertility, early age at first birth, early menopause, HRT treatment, and hysterectomy.

This study was cross-sectional and, therefore, unable to assess the impact of pregnancy loss on cardiac geometry over time, with varying time periods between the pregnancy loss and our assessment of participants. Finally, no information was available on other pregnancy complications linked to future CVD such as hypertensive disorders of pregnancy, preterm birth and gestational diabetes, so we cannot comment on other possible explanations for the known link between pregnancy loss and future CVD risk.

Despite these limitations, a broad heterogeneity of socioeconomic and ethnic groups, in a large study population, in which a range of potential confounders have been measured and gold-standard measures of subclinical CVD are available should increase confidence that valid scientific inferences can be drawn from the lack of association between a history of pregnancy loss and cardiac remodeling, which is likely to be generalisable to the wider population.

## Conclusion

Women who self-report pregnancy loss do not have significant differences in cardiac structure, cardiac function, or carotid structure in later life to explain their increased cardiovascular risk. This suggests any cardiovascular risks associated with pregnancy loss operate through other disease mechanisms. Alternatively, other characteristics of pregnancy loss, which we were not able to take account of, such as timing and number of pregnancy losses may be required to identify those at greatest cardiovascular risk.
